# Case studies for implementing MCDA for tender and purchasing decisions in hospitals in Indonesia and Thailand

**DOI:** 10.1186/s40545-021-00333-8

**Published:** 2021-06-14

**Authors:** Anke-Peggy Holtorf, Erna Kristin, Anunchai Assamawakin, Nilawan Upakdee, Rina Indrianti, Napassorn Apinchonbancha

**Affiliations:** 1 Health Outcomes Strategies GmbH, Colmarerstr 58, 4055 Basel, Switzerland; 2 College of Pharmacy, Pharmacotherapy Outcomes Research Center, University of Utah, Salt Lake City, UT USA; 3 Department of Pharmacology Therapy, Faculty of Medicine, Public Health and Nursing, Universitas Gadjah Mada, Yogyakarta, Indonesia; 4 Department of Pharmacology, Faculty of Pharmacy, Mahidol University, 447 Sri-ayuthaya Road, Rajathevi, Bangkok, 10400 Thailand; 5 Faculty of Pharmaceutical Sciences, Naresuan University, Phitsanulok, 65000 Thailand; 6 PT. Abbott Indonesia, Wisma Pondok Indah 2, Suite 503, Jalan Sultan Iskandar Muda Kav V/ TA., Pondok Indah, Jakarta Selatan, 12310 Indonesia; 7 Abbott Laboratories ltd, No.1, Q House Lumpini, 30th & 33rd Floor, South Sathorn Road, Thungmahamek, Sathorn, Bangkok, 10120 Thailand

**Keywords:** Multi-criteria decision analysis, MCDA, Multi-source pharmaceuticals, Tender selection, Purchasing, Implementation, Hospital pharmacy, Hospital formulary, Low- and middle-income countries, LMICs

## Abstract

**Background:**

A multi-criteria decision analysis (MCDA) approach has been suggested for helping purchasers in low- and middle-income countries in an evidence-based assessment of multi-source pharmaceuticals to mitigate potential adverse consequences of price-based decisions on patient access to effective medicines. Six workshops for developing MCDA-instruments for purchasing were conducted in Indonesia, Kazakhstan, Thailand, and Kuwait in 2017–2020. In Indonesia and Thailand, two pilot-initiatives aimed to implement the instruments for hospital drug purchasing decisions.

**Objective:**

By analysing and comparing the experiences and progress from the MCDA-workshops and the two case-examples for hospital implementation in Indonesia and Thailand, we aim to gain insights, which will support future implementation.

**Methods:**

The selection of criteria and their average weight were compared quantitatively across the MCDA-instruments developed in all four countries and settings. Implementation experiences from two case-examples were studied, which included (1) testing the instrument across a variety of drugs in seven hospitals in Thailand and (2) implementation in one specialty hospital in Indonesia. Semi-structured interviews were conducted via web-conferences with four diverse stakeholders in the pilot implementation projects in Thailand and Indonesia. The open responses were evaluated through qualitative content analysis and synthesis using grounded theory coding.

**Results:**

Drivers for implementation were making ‘better’ decisions, achieving transparency and a rational selection process, reducing drug shortages, and assuring consistent quality. Challenges were seen on the technical level (definition or of criteria, scoring methods, access to data) or change-related challenges (resistance, perception of increased workload, lack of competencies or capabilities, lack of resources). The comparison of the MCDA instruments revealed high similarity, but also clear need for local adaptations in each specific case.

**Conclusion:**

A set a of measures targeting challenges related to utility, methodology, data requirements, capacity building and training as well as the broader societal impact can help to overcome challenges in the implementation. Careful planning of implementation and organizational change is recommended for ensuring commitment and fit to local context and culture. Designing a collaborative change program for each application of MCDA-based purchasing will enable healthcare stakeholders to maximally benefit in terms of quality and effectiveness of care and access for patients.

**Supplementary Information:**

The online version contains supplementary material available at 10.1186/s40545-021-00333-8.

## Background

In low- and middle-income countries (LMICs) purchasing and tender decisions for multi-source pharmaceuticals (off-patent pharmaceuticals supplied by two or more sources) are often driven by price only [1]. However, that can have detrimental impact as other important factors are not considered, such as product quality, production quality, or ability to deliver. Inferiority in any of these aspects may reduce benefits and increase the risk for harms to patients due to compromises in, for example, patient safety [2] or increased occurrence of drug shortages [3, 4].

Multiple Criteria Decision Analysis (MCDA) is “an umbrella term to describe a collection of formal approaches which seek to take explicit account of multiple criteria in helping individuals or groups exploring decisions that matter” [5] and it has been suggested to use an MCDA approach for evidence-based assessment of multi-source pharmaceuticals in developing countries [6, 7]. MCDA appears suitable in helping to consider multiple and sometimes conflicting criteria in the evaluation of the available alternatives [8].

Following initial introductory and MCDA development workshops in Indonesia [9], Kazakhstan [10], Kuwait [11], and Thailand [12], the implementation guidance suggested well documented piloting projects as first steps for implementation in a country [13]. Academic project champions in Indonesia and Thailand initiated such pilot projects as case examples in their respective environments in partnership with a national pharmaceutical association.

In Indonesia, Hospital formularies generally follow the National Formulary (Fornas) and use an e-catalogue for procurement, especially in hospitals serving the patients covered by Badan Penyelenggara Jaminan Sosial (BPJS; Social Insurance Administration Organization that administers the Indonesian national health insurance Jaminan Kesehatan Nasional, JKN). The specific products in the formulary in each hospital are currently selected based on active pharmaceutical ingredient (API) and the lowest bidding price, but hospitals frequently experience drug shortages and compromises in quality. A pilot project for implementing MCDA was set up for hospital formulary decisions and procurement at the National Brain Center Hospital (PON Hospital, Jarkata) to test applicability in the hospital environment [14] with the primary goal to reduce the occurrence of drug shortages and to ensure a consistent and reasonable level of quality.

Before 2017, the single selection criterion in tenders or biddings was the lowest price in Thailand. Since the establishment of the Public Procurement Government Procurement and Supplies Management Act, B.E. 2560 (AD 2017), the bidder selection for multi-sourced supplies, including pharmaceuticals and medical products, has been expanded beyond “price” to “price-performance” in order to align with the principles of the Act concerning worthiness, transparency, efficiency, effectiveness and accountability. Public hospitals are encouraged to use performance criteria to determine the suppliers for pharmaceutical products. However, there is still a lack of a standard definition of what performance criteria encompass and how important each of them is in making the decision. The consequence is a high level of variation between the hospitals in the formulary composition and in the methods shaping the specific bidding process. To improve quality and transparency, the government is newly requiring a solid rational and transparent documentation of hospital purchasing decisions. A pilot MCDA project starting in 2019 aimed at testing the feasibility of using MCDA in the decentralized purchasing of hospital pharmaceuticals [15]. The motivation of participating in the pilot implementation project in Thailand was to meet the governmental requirements for consistency and transparency through harmonizing the decision approach across the seven participating hospitals.

By analysing and comparing the experiences and progress from the MCDA-workshops described above and, more in-depth, the experiences with the case examples in Indonesia and Thailand, we aimed to gain insights for improving future implementation in Thailand and Indonesia and, potentially, in other LMICS.

## Methods

Six MCDA consensus workshops following the same guidance and principles [13] were held in Indonesia, Thailand, Kazakhstan, and Kuwait between 2017 and 2020 and the MCDA instruments resulting from these workshops have been published [9–12]. In both Thailand and Indonesia, the workshops resulted in initiatives for implementing the method on a broader basis, starting with a pilot (case study) in each country. To analyse the experiences of core stakeholders in the two case studies relating to the implementation of MCDA-supported formulary decisions in hospitals, four stakeholders were interviewed for each case study. The interviews were evaluated through qualitative content analysis and synthesis following published guidance [16, 17]. The interview process is described in “Interviews for pilot implementation in Thailand and Indonesia” section.

One question for future application of an MCDA-based decision process in the acquisition of multi-source products was, whether a standardized instrument can be used across countries rather than bespoke instruments. To test this option, the MCDA-models and instruments resulting from all MCDA workshops in Indonesia, Thailand, Kazakhstan, and Kuwait were compared as described in “Comparison of MCDA tools” section.

**Fig. 1:**
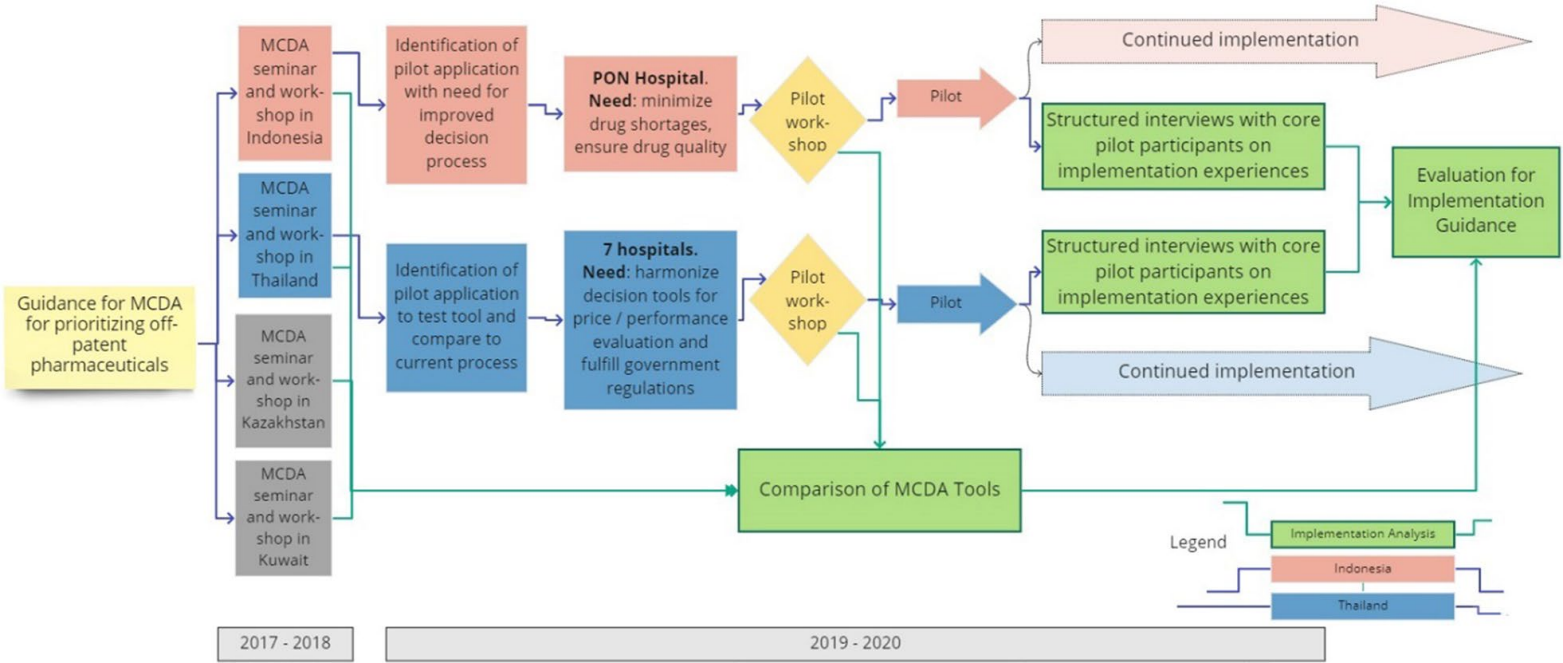
Graphical study flow. Graphical overview on the organization of MCDA workshops and pilot studies, which formed the basis for this report. The parts relating to the implementation analysis are coloured green

An overview of the entire flow of events is shown graphically in Fig. 1. The parts in green colour are reported in this study.

### Interviews for pilot implementation in Thailand and Indonesia

#### Interviews

An interview questionnaire was developed and tested with an industry stakeholder in Thailand and Indonesia, who had been engaged throughout the projects in their respective country. Thereafter, the final 16-item questionnaire (see Additional file 1) guided interviews with 3 diverse non-industry stakeholders (academic, hospital pharmacists, hospital administration) involved in each of the implementation projects. The experience and expectations of the interviewees were explored as well as the facilitating or inhibiting factors, barriers and stakeholders to be involved in the implementation. Except one question requiring the rating of 9 potential barriers between 1 (no barrier) and 5 (very high barrier), all other questions were open ended and did not prompt any answers.

Interviewees were selected for their familiarity (duration and intensity of being involved in the implementation) with the respective pilot project and to reflect different perspectives. The questionnaire was shared with the interviewees at least 3 days before the interview. The 45–60-min interviews were conducted via web-conference (GoToMeeting) in October/November 2020 in English by one interviewer (AP Holtorf, PhD, MBA, female) who had not been involved in either of the pilot projects. At the beginning of each interview, the purpose of the interview was explained and the willingness to participate confirmed. Notes were taken during the interview while sharing the screen, and the discussion was recorded. Afterwards, the notes were revised with the help of the recording and shared back with the interviewee to allow for corrections or additions.

The evaluation included qualitative content analysis [16, 17] and synthesis and was performed consistently by one evaluator. A narrative summary table was produced for each of the pilots including the answers from all interviewees (*n* = 4 for both hospital pilots in Thailand and Indonesia). The average rating of barriers was calculated per implementation project in a spreadsheet program, and the following responses were coded guided by the items mentioned by the interviewees (grounded theory coding [18]): accelerating and inhibiting factors, stakeholders, successes, and difficulties encountered throughout the process. Opportunities and challenges were reported using an axial coding approach [19] following the categories reported previously by the FIFARMA (Latin American Federation of Pharmaceutical Industry) MCDA taskforce [20]: utility, methodology, data requirements, capacity or training requirements, or broader societal impact.

### Comparison of MCDA tools

For each workshop, there was a preparatory phase including the preliminary selection and adaptation of the criteria and scoring starting from a list proposed internationally for the evaluation of multi-source pharmaceuticals [6] to the specific decision context (e.g., national tender, hospital formulary, etc.). Subsequently, important stakeholders in the decision were invited for one or two days of workshop depending on the expected level of pre-existing knowledge and need for introductory training. For the Thai hospital pilot project, each workshop was conducted in one day. Two workshop days were offered in the Indonesian pilot: the first day in October 2019 for training and for initiating the hospital specific data collection to support the project, and the second day in March 2020 for the collaborative development of the MCDA tool, as described subsequently. The core part of the workshop guided the participants through five interactive steps for local adaptation of the previously developed and validated global MCDA format: (1) criteria selection; (2) scoring definition; (3) weighting of price criterion; (4) definition of cut-off point for price criterion; (5) ranking and weighting of remaining criteria. The detail of this process is described elsewhere [9, 11–13]. As an exception, in the hospital workshop in Indonesia (in 2020), ranking and weighting happened through focus groups and consensus discussions. All consensus judgements were imported to a validated spreadsheet decision instrument (Excel® spreadsheet) suitable for being used in the real-world situation to support the decision and to document the rationale behind the decision. Each workshop ended with a moderated discussion, in which the participants agreed on next steps towards piloting the resulting MCDA tool. The final instruments, the criteria and weighting of all MCDA tools resulting from the workshops were imported into one summary table to allow the comparison across the different workshops.

## Results

### Status of the implementation projects

#### Indonesia hospital drug purchasing

The National Brain Center Hospital (PON Hospital) was selected for the pilot implementation of MCDA in purchasing of multi-source drugs. A preparatory workshop for training and for initiating data collection was held in October 2019 followed by an MCDA consensus workshop in February 2020. The 17 participating hospital stakeholders included personnel from the hospital administration, departmental directors, hospital pharmacy and clinical pharmacists, public relations, internal auditing, clinical care, surgery, and neurology [14]. The workshop resulted in a consensus on MCDA criteria, scoring definitions, ranking and weighting (for more detail, see ‘Indonesia, Hospital’ in Fig. 3) and a hospital specific MCDA instrument, which was supposed to be tested subsequently in real-life purchases of multi-source pharmaceuticals. This step has been postponed due to the world-wide COVID-19 pandemic. A regulatory trial in the drug selection process for the hospital’s formulary will be conducted throughout 2021 for the prioritization of multi-source products with the same active pharmaceutical ingredient.

#### Thailand hospital drug purchasing

After an initial MCDA design workshop with 37 multi-stakeholder participants in June 2018 [12] for creating the MCDA tool, a second MCDA workshop was held with a group of 10 representatives from 7 selected pilot hospitals in September 2019 facilitated by the Pharmaceutical Association of Thailand under the Royal Patronage. The goal of the second workshop was to initiate a pilot application of the MCDA tool in a real-life drug evaluation with purchasing pharmacists of the seven hospitals. In each hospital, three of ten possible drug purchasing decisions were performed in neurosurgery, chronic diseases, anti-sepsis, or Cox-II-inhibitors using the previously developed MCDA and the decisions were compared to the decisions under the previous price-performance decision principles. The detailed data collected during the pilot phase were reported [15].

The evaluations were performed within the seven hospitals between March and September 2020. The pharmacists participating in this pilot for price-performance evaluation through MCDA were satisfied with the criteria and considered the criteria selection as an improvement [15].

### Interviews with key stakeholders

Eight interviews were conducted in total, including three with leading participants in each of the two hospital case studies and the two pilot interviews with industry stakeholders. While the full summary tables resulting from the interviews are available as supplementary online material (Additional file 2, Additional file 3), key findings are reported below.

#### Drivers for introducing MCDA

As reasons for using MCDA in their specific decision context, the interviewees listed a range of drivers including the prospect of making ‘better’ decisions, achieving transparency and a rational selection process. While in Thailand, consistency across decisions between hospitals was mentioned as an important driver, prioritizing the specific needs of the hospital was mentioned as advantage in Indonesia (see Table 1 “Rationale for using MCDA”).

**Table 1:** Interview responses part 1

	Thailand hospital tender (*n* = 4)	Indonesia hospital drug purchasing (*n* = 4)
Rationale for using MCDA	Improve quality at reasonable cost Achieve transparency with agreed criteria Consistency across hospitals	Listing only the best (most effective and affordable) alternatives Rational selection process Decisions meet hospital needs
Opportunities: expected differences due to MCDA (number of mentions)	Better patient health [3] Transparency [2] Better accuracy/consistency in scoring [2] Better acceptance of purchasing decision by HCP and patients [2] Quality improvement [1] More fairness in tender [1] Change in hospital purchasing practices [1] Better product performance [1] Awareness [1] Better guidance of companies what type of products are expected [1] Better access for high-quality products [1]	Quality of products [4] Affordability [2] More objective/rational decision [2] Transparency in tender or formulary selection [2] Fairness in Off-Patent-Pharmaceutical market [1] Better decisions on the formulary [1] Better (monitoring of) availability [1] More (objective) consideration of value-added services [1]

Summary of interview responses relating to the rationale for using MCDA in the respective decision process and the expected improvements (differences), which could be achieved

The list of opportunities was much longer (see Table 1 “Opportunities/expected differences”). Frequently mentioned items were ‘better patient health’, ‘quality of products’, ‘transparency’, ‘consistency’, and other items like ‘fairness in the market’ or ‘better acceptance of the decisions’.

#### Barrier ranking

**Fig. 2:**
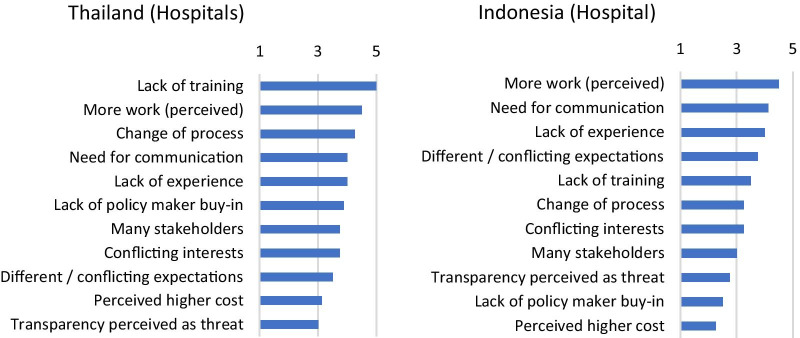
Implementation barriers. Ranking of common implementation barriers as perceived by the interviewees. The interviewees rated the pre-determined barriers between 1 (no barrier) and 5 (high barrier)

When asked to rate the importance of 11 pre-determined potential barriers, the highest-ranking barriers across the hospital settings in Indonesia and Thailand were ‘More work (perceived)’, ‘Lack of experience’, ‘Need for communication’, and ‘Lack of training’ as well as ‘Change of process’ in Thailand and ‘Different/conflicting expectations’ in Indonesia. The ratings of the barriers by the interviewees from Indonesia and Thailand are shown in Fig. 2. On the other side, the risk that transparency might be perceived as a threat was rated comparably low in both hospital-pilots but still with an average rating of around three.

It is important to note that the perceived importance of the barriers in both pilots shows distinct differences. For example, ‘Different/conflicting expectations’ and ‘Transparency perceived as threat’ ranked much higher in Indonesia than in Thailand, while in Thailand, ‘Change of process’ ranked higher.

#### Successes and difficulties

Most interviewees reported that the communication and involvement of the stakeholder, creating awareness about the issues and possible solutions, and attaining their interest went very well (see Tables 2 and 3, Successes).

**Table 2:** Interview responses part 2

	Thailand hospital tender (*n* = 4)	Indonesia hospital drug purchasing (*n* = 4)
Successes (what went well?) (number of mentions)	Good communication and collaboration between. project leaders and hospitals [2] MCDA adoption [1] Participant motivation [1] Fits into current process [1]	High interest in hospital [3] Creating awareness [2] Stakeholder involvement [1] Using local data [1]
Challenges (Difficulties) (number of mentions)	Assessing the criteria (all 4) Resistance to change [2] Standardizing the scoring [1] Need to improve criteria [1] Practice integration [1] Need for training [1]	Finding data for scoring [2] Perceived as more complex and more work burden [2] Pandemic interrupted the initiative [2] Drug shortages can have other reasons [1]

Summary of interview responses relating to successes and challenges (difficulties) in the implementation projects

**Table 3:** Interview responses part 3

	Thailand hospital tender (*n* = 4)	Indonesia hospital drug purchasing (*n* = 4)
Accelerators (number of mentions)	Education in evaluation techniques [3] National policy [3] Support/advice [1] Clarity of criteria [1] Simple Tools [1]	Training [3] Leadership support [2] Practical experience [1] Association support (Medical and Hospital) [1] Using local hospital data [1] Solve conflicts in interest [1] Access to data [1]
Inhibitors (number of mentions)	Complexity of assessing criteria (all 4) Resistance to change [2] Lack of scoring definition [1] Subjectivity of some criteria [1] Practice integration [1] Need for training [1]	Lack of training and knowledge [3] Conflicts with current process [2] Industry rejection [1] Complexity of MCDA process [1]
Stakeholders (the ones who were involved are in bold type fonts; the other were also mentioned as important)	**Academic pharmacists** **Hospital pharmacists** **Head of hosp. pharmacy** **Head of specialty department for PTC** **Director of hospital** **Procurement pharmacists** Prescribers/Clinicians Pharmaceutical industry Finance	**Academic leaders** **Hospital director** **Hospital administration** **Clinicians/Prescribers** **Pharmacists (supply manager)** **Therapeutic committee** Hospital association National Agency of Drug and Food Control of Republic of Indonesia Pharmaceutical companies Financial and legal department of hospital Patient organization
Potential role of PAGs/POs	Advocate for the importance of product quality and high-quality products Increase awareness for the issue Patient consensus on criteria	Analysis of patient experiences to create awareness for issue Concern: potential lack of objectivity

*MCDA* Multi-Criteria Decision Analysis, *MoH*   Ministry of Health, *PAG* Patient Advocacy Group, *PO *Patient Organization, *PTC* Pharmacy and Therapeutics Committee

Summary of interview responses relating to accelerators or inhibitors observed in the implementation project so far and the potential role of patient advocacy groups (PAGs)

On the other hand, several challenges were identified, which included technical challenges (assessing the criteria, scoring standards), challenges related to change (resistance, practice integration), and challenges related to the users (need for training, perception of additional work and complexity).

#### Perceived accelerators, inhibitors and stakeholders

The interviewees across the board identified training, education, and practical experience with MCDA methodologies or relating to the specific evaluation techniques as effective means for accelerating the implementation. Additional items were suggested, as can be seen in Table 3 (in row Accelerators) such as leadership support, association support, using local data in Indonesia, and a mandate through a national policy and tools or national support for evaluating the criteria in Thailand.

In contrast, a few items were expected to hinder implementation (Table 3, in row Inhibitors): complexity of criteria assessment or scoring and the difficulty to integrate MCDA with the current decision pathways and regulations.

A perceived risk was that some suppliers could reject the new process. The new process could be a threat for those suppliers with products that only meet the minimal scoring, who do not have sufficient evidence supporting the performance of their products, or who have frequent supply issues.

All interviewees mentioned a range of stakeholders who should be involved as listed in Table 3 (in row *Stakeholders*). For both hospital projects, the participation was mostly restricted to a range of hospital personnel including administrative leadership and academic advisors. The interviewees mentioned a few others who had not been involved (Table 3, Stakeholders) printed in regular font).

Some interviewees thought that patient advocacy groups (PAGs) could play an important role in increasing the awareness of the issues but would require some training and education to be able to do so. One interviewee suggested that PAGs could also help in the initial framing of the issues by outlining their experiences with the current access to medicines and choices. On the other hand, there was also a concern mentioned, that patient involvement would have to happen carefully to avoid undesirable impact on the decision process.

### Comparison of MCDA ranking and weighting

**Fig. 3:**
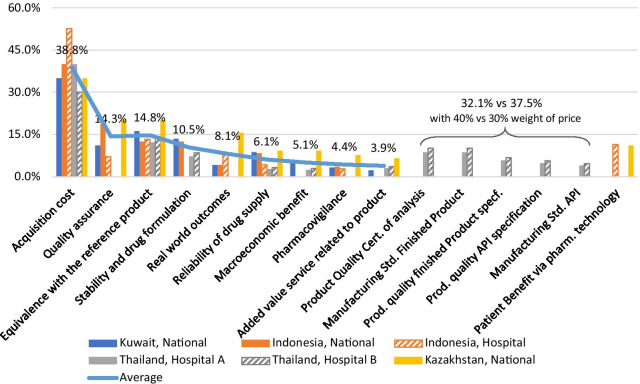
Comparison of MCDA tools across countries. Summary results from MCDA consensus workshops in Indonesia, Kazakhstan, Kuwait, and Thailand. The numbers indicate the average weight (in percent) across the different workshops

Figure 3 displays the results for the MCDA ranking and weighting derived from all workshops conducted between 2017 and 2020 in four countries. The consensus criteria chosen by the participants resembled mostly the criteria originally suggested by Brixner et al. [6]. The resulting MCDA tools included between seven (Indonesia, national and hospital level) and 11 (Thailand, hospital level) criteria.

The weight of the price criterion in the final decision varied between 30% (Thailand, selected pilot hospitals) to 52.5% (Indonesia, in pilot hospital). Quality assurance and Equivalence with the reference drug were deemed the next most important criteria, while ‘Macroeconomic benefit’, ‘Pharmacovigilance’, and ‘Added value services related to product’ usually ranked lowest or were not included.

While all other countries used a summary criterion ‘Quality assurance’, the Thai stakeholders selected 5 different criteria to give—as a group—a more granular reflection of quality and resembling the current priority on quality improvement. In two cases, Kazakhstan and the Indonesian hospital case example, an additional criterion “Patient Benefit via pharmaceutical technology’ were added with a weight of 11.1 or 11.4%, respectively.

## Discussion

The decision on the drug formulary and which type of medicines are available to the patients has a high impact on the health of the majority of patients in LMICs [21]. When only prioritizing a formulary by price, the total cost of procuring and using a product is forgotten, meaning that potential cost consequences of safety issues (adverse events), drug shortages (i.e., no access of treatment for patients or only at much higher acquisition cost and work), or other shortcomings (e.g., lack of pharmacovigilance or other support) would not be considered. Using multiple criteria in the selection process may help to prioritize access to effective medicines and consequently, to improve the health of the patients. Selecting drugs for the formulary or drug list through MCDA could therefore present a useful approach towards making more rational, more sustainable choices in the interest of the patients and the healthcare system. Examples for using quantitative MCDA approaches [22] in listing, purchasing, tendering, or pricing decisions for commonly used medicines have been published including those which formed the base for the implementation project reported in this paper [9–12] and others [23–26]. Here we focus on the question how to implement this new, MCDA-based decision process in hospital purchasing at the example of two case studies in Indonesia and Thailand.

### Implementing MCDA starts with a need

The ISPOR MCDA Emerging Good Practices Task Force suggests in their guidance to start by defining the objective to be achieved with the MCDA [5, 27]. This is important for both developing the MCDA instrument and planning the implementation in the respective setting of use.

In our study, we learned that making better decisions, achieving transparency, consistency, and a rational selection process were mentioned as main drivers for the hospital-pilots in Thailand and Indonesia. In Thailand, this need was mostly founded in the national policy priorities. In Indonesia, the needs were also strongly driven by the pre-workshop analysis of the hospital’s data relating to the purchasing and performance of the products in question. In other cases, specific dysfunctions ought to be addressed or there may be a need to adapt current processes to new policy priorities.

As outlined by Baltussen et al., all of these objectives can be achieved through using MCDA which fosters in-depth and evidence-based consideration of explicit criteria [22]. Using the same set of explicit criteria repeatedly across evaluations improves the consistency of the resulting recommendations. Using explicit criteria and documenting the evaluation process transparency is supported by publishing the criteria and process openly or at least to those concerned by the decision [22]. Concrete challenges such as drug shortages or inferior product-quality can be addressed directly by testing for the performance against criteria targeting these issues.

Once the objectives for adopting an MCDA approach have been defined, it is also important to increase the awareness of the underlying issues with all stakeholders, which can happen through targeted information, through publications, advocacy, or individual discussions. The expected differences listed in Table 1 (in row Expected differences) support these objectives and could help to inform potential monitoring instruments.

### Generalization versus tailoring of MCDA tools from different environments

The easiest for local or national users of an MCDA instrument would be to adopt a validated standard instrument. To understand the degree of similarity or interchangeability between the instruments developed by the same methods in different contexts [13], we compared the MCDA algorithms developed in different countries or healthcare contexts between 2017 and 2020: in Kazakhstan, Indonesia, Kuwait for drug purchasing decisions concerning a population/national level, and in Indonesia and Thailand on a hospital level [9–12]. This comparison (see Fig. 3) led to several observations.

#### Selection of criteria

Most of the international baseline criteria described by Brixner et al. in 2017 [6] were maintained with slight variations (adding or excluding criteria). Specific criteria were excluded for reasons related to the feasibility of obtaining the data, or because (perceived) irrelevance of the criterion in the context. Criteria were added to address specific issues. For example, in Thailand, five new criteria replaced ‘Quality Assurance’ resulting in an overall decision weight for quality of 32.1–37.5%. Both in Kazakhstan and the Indonesia hospital pilot, a criterion ‘Patient benefit via pharmaceutical technology’ was added and given a similar weight of 11.1 or 11.4% in the overall decision.

#### Weight of price criterion

The weight of the price criterion in the final decision ranged between 30 and 50 percent. Two of the hospitals participating in the case study in Thailand, decided to use lower weight for the price criterion (30%) than the rest of the hospitals. This indicates that there are different value perceptions and expectations or ‘cultures’ existing across different hospitals.

#### Relative weight of non-price criteria

The most important non-price criteria were *Quality assurance*, *Equivalence with the reference product*, and—in those environments, where drug shortages impede clinical–pharmaceutical care, *Reliability of drug supply*, with weights ranging between 6.4 and 20.2% (except for Thailand with a combined weight of 32.1% or 37.5% for the quality criteria).

#### Transferability of criteria and weights

While in principle, the criteria seemed transferable across different countries and contexts, the priorities and weightings varied in the different MCDA algorithms. If consistency of decision processes across institutions is important, alignment across institutions must be achieved. However, if targeting the decision priorities to the needs in the specific context of an organization is important, the instrument must be tailored for the individual organization.

A report from Iran on using an MCDA approach to select ‘best buys’ in non-communicable diseases, concluded that the more data were available for each of the alternatives to be evaluated, the more comfortable the authors were with the reliability and robustness of the decision [23]. Hence, even if some principles of the MCDA approach and the basic set of criteria can be transferred across settings or boarders for similar decision problems, it is always important to ensure that the criteria and scoring fit the local context and the availability of data at the point of decision.

Furthermore, local adaptation allows for participation of local stakeholders, which is critical in incorporating societal values, preferences, or practices as reported for other decision problems such as evaluation of orphan drugs [28]. In addition, the multi-stakeholder creation process helps to create understanding, acceptance, and ownership among the local stakeholders (appropriation), and hence, it is an important step in the implementation process.

### Successes, strengths, and challenges in implementation in Indonesia and Thailand

All interviewees reported successes, which mostly related to communication among the stakeholders, increased awareness of the issues, and high interest in the applying the method in the respective environment. One interviewee from Indonesia reported for example, that although the tool was not yet adopted to routine evaluation in the hospital, the participating pharmacists had already started to ask more critical questions relating to the criteria to their suppliers.

These responses are well aligned with an example of using an MCDA approach for supporting reimbursement decisions in a province in Canada, where the authors report successful implementation thanks to: good communication level between MoH and pilot institution, shared vision, champions and allies, clear need and objective (fixed budget, risk of trade-offs with equity), set within the process and not as replacement of process, and minimal policy change required [29].

On the other hand, the implementors in our study also identified challenges, mostly related to technical issues (criteria definition, data retrieval, and scoring) and issues related to change management (resistance to change, practice integration).

In barrier rating, the interviewees indicated that the perception that the new process would likely lead to *much more work for the users*, *lack of experience and training*, the *high need for communication with all stakeholders* potentially affected by the new decision process, *conflicting expectations*, and the* change of process* were identified as the highest barriers by the hospital implementors. These barriers confirm observations in other countries. For example, an expert panel in another Canadian initiative expected the biggest barriers for implementing MCDA for HTA in fragmented decisions, conflicting perspectives, complexity, budget constraints, and lack of political will [30]. However, differences could be seen between the case studies in the two countries as well as how the barriers were perceived by different stakeholders. Therefore, it is important to analyse the barriers in each implementation case to be able to specifically address those barriers which are most critical in the local context.

We categorized the findings from the interviews in a matrix of opportunities (or strengths) and challenges relating to utility, methodology, data requirements, capacity and training requirements, and broader societal impact with MCDA as shown in Table 4. The high level of interest achieved in the hospitals and, to some degree, on the policy level in combination with the reported strengths and opportunities suggests, that there is generally a positive environment and demand for MCDA. However, the challenges need to be addressed to overcome the resistance to change. The ‘Recommendations’ listed in Table 4 should be understood as examples of actions which could be taken to overcome the challenges and they include open communication, information, and education as well as ensuring that the criteria and evaluation techniques are relevant and feasible in the respective context.

**Table 4:** Opportunities and challenges

	Strengths/opportunities	Challenges	Recommendations
Utility	Transparency Adaptability to local priorities/needs Participatory and multi-stakeholder approach Consideration of trade-offs (price vs. quality + non-quality criteria)	Requires replacement of current process Need for evidence to prove the MCDA effectiveness Perception of complexity in implementation Requires more time and work than price-only decisions	Create full transparency Involve broadly throughout process Support and reinforce new behaviour Measure success
Methodology	Consistent/systematic decision approach Process quality assurance Flexibility (e.g., different value frameworks for different drug classes) Pragmatic, user-oriented, and modular	Criteria selection, validation, and measurement; improve applicability for users Criteria definitions need to be more precise	Ensure fit of criteria in evaluation setting Detailed guidance on criteria and scoring
Data requirements	Adaptable to local data	Data retrieval and synthesis by criteria Data interpretation	Detailed guidance on data requirements
Capacity/training requirements	Upskilling of evaluating personnel	Lack of training and knowledge Need for continuous re-enforcement	Education on why Training on how Retrain
Broader societal impact	Criteria as requirements to be met by suppliers upskilling of suppliers	Threat perceived by local industry	Guidance for manufacturers/suppliers Self-scoring tools Feed-back to suppliers Training for suppliers Full transparency

Analysis of strengths or opportunities versus challenges as observes in the pilot implementation projects in Thailand and Indonesia, following the model used by the FIFARMA MCDA taskforce (Latin American Federation of Pharmaceutical Industry) [20]. The third column (Recommendations; with grey background) exemplifies actions to realize the opportunities and to address the challenges

A reference should be made to the concept of ‘sticky knowledge’ as model for embracing change or barriers to change in healthcare [31, 32]. Multiple factors are described in the sticky knowledge model which are relevant for context of implementing MCDA in healthcare decision-making: causal ambiguity, unproven knowledge, motivation of source, credibility of source, recipient motivation, recipient absorptive capacity, recipient retentive capacity, barren organizational context, and arduous relationship between source and recipient [32]. Some of these factors have apparently been addressed quite well in the implementation projects described in our paper (e.g., causal clarity, evidence, academic leaders driving the projects) while others may need more attention (e.g., recipient absorptive capacity, recipient retentive capacity, barren organizational context).

Several interviewees suggested to better define the criteria and to simplify data retrieval and interpretation for the evaluators. Insufficient definition and guidance may leave too much room for interpretation and consequently, a lower consistency across different users [33]. For example, ‘Macroeconomic benefit’ is difficult to assess for a hospital pharmacist. Also, the concept of ‘Bioequivalence’ was not very well known and therefore not understood by some users. Therefore, before using such tools on a broader level, each of the criteria must be well defined including appropriate cut-off ranges and scoring methods, and the users must be sufficiently trained. Alternatively, as suggested by some of the Thai interviewees, more high-level criteria could be evaluated on a national level and made available to the local evaluators, while these locally assess those criteria, where local information and value context is required.

In both hospital-pilots described in this paper, concerns were also mentioned towards complex work involved in the retrieval of data. This is an important aspect, which must be addressed in the design phase. It needs to be clear to the evaluators where they can find the data and how to interpret them. To facilitate this aspect, an international group of health policy experts has developed an ‘Evidence Framework for Off-Patent Pharmaceutical Review’ (EFOR) to enable evaluators to standardize the evaluation and to enable suppliers to deliver the best available evidence for their product relating to each criterion.[34] Following this international example, a guidance could be elaborated and shared with the suppliers. In Qatar, this approach was chosen for the prioritization of proton pump inhibitors using an MCDA approach: The companies responsible for marketing and distribution of the product in Qatar were contacted to supply the information and the supportive published evidence.[26] The objective was not that suppliers made the evaluation themselves but to give all companies the opportunity to supply the best possible evidence for their product. However, a self-evaluation checklist summarizing the submitted evidence could facilitate the orientation and retrieval of the information relevant to each criterion. It could also help to increase the awareness of the suppliers which criteria are relevant for the selection and what data are needed in support of future submissions.

### Towards a best practice implementation framework for MCDA

Implementation requires systematic facilitation of the organizational change and creating a supportive culture throughout the implementation process. How to evoke change in organizations has been extensively studied. The gold standard was defined as an eight-item circuit by Kotter in 1995 [35], and in subsequent work, named the eight ‘accelerators’ [36]: establishing a sense of urgency, creating a guiding coalition, developing a change vision, communicating the vision for buy-in, empowering broad-based action, generating short-term wins, never letting up, and incorporating changes into the culture [35, 36].

What is needed for more effective implementation of the MCDA framework? We propose actions on each level of Kotter’s eight accelerators, formed with implementation measures derived from the accelerators and inhibitors proposed by the interviewees based on their experiences with the pilot projects.

**Fig. 4:**
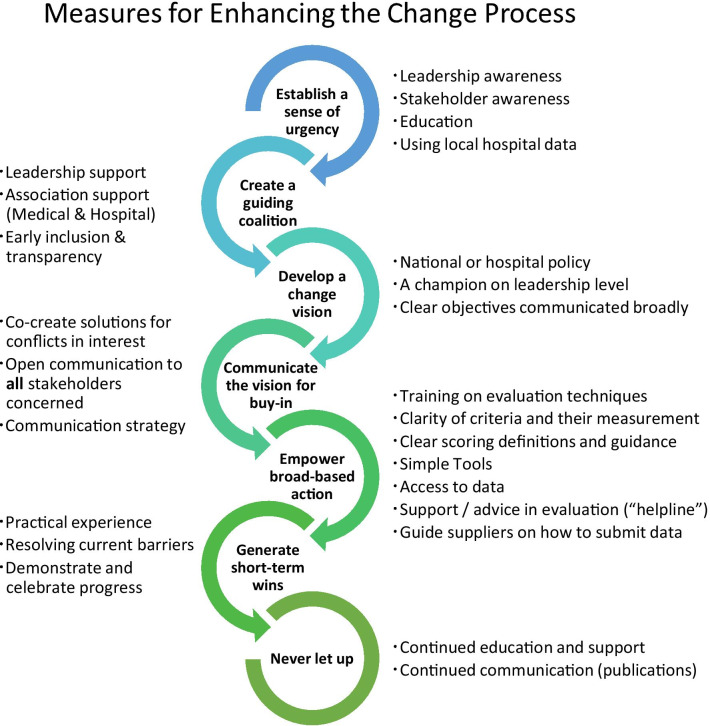
Change management for implementation. Measures to enhance implementation along the change management accelerators according to Kotter [36]

The resulting implementation framework is presented in Fig. 4 and may help guiding implementation leaders in designing their local targeted implementation plan.

Concurrent action, well adapted to the scope of the implementation and the local context, across all eight change accelerators while rapidly building a network of change agents should maximize its adoption and impact [36].

Adaptation to the local context depends on access to and inclusion of the key stakeholders and influencers within the own healthcare environment. A core group of such stakeholders has been involved in both implementation projects described in this study. However, during the interviews, additional stakeholders were mentioned who could have been relevant but had not been involved. Although most of the interviewees thought, that patient organizations could be useful in some important elements such as increasing awareness of the other stakeholders and advocating for basic patient rights, no patients had been involved up to date.

Finally, it should be re-emphasized, that the plan for implementation cannot be a standardized template. A Canadian research team concluded from a study of priority setting best practices in healthcare organizations in Canada that not only quantifiable performance facts foster implementation, but how these are woven into the organization and its culture, and how that this is translated in to addressing challenges, setting priorities, and allocating resources [37].

Hence, while implementation of MCDA should be done strategically with consideration of all ‘accelerators’ proposed by Kotter, it is important to strongly connect the actions to the local needs, ecosystem, and cultural context, to involve all stakeholders affected by the decision, and to maintain transparency and willingness to improve throughout the journey.

### Limitations

Among the limitations of this work is firstly, that only 4 people were interviewed for each implementation project, mostly members of the respective leadership group. Therefore, certain perspectives of other stakeholders may have been missing. Nevertheless, several view-points were covered including academic experts in health policy, pharmacy management or pharmacy practice, users from the pharmacy practice, a hospital leader, and industry representatives. In future research, an additional source of information to better understand and address the barriers could be to interview those who oppose or resist against the new decision process. In the current study, we have only interviewed supporters of the MCDA process.

Another important limitation is that the Indonesian implementation project was not yet completed at the time of writing this manuscript due to being interrupted by the global events of the COVID-19 pandemic, which changed the hospital priorities foregoing the opportunity to use the MCDA process already in this crisis to strengthen the hospital’s decision procedures in drug purchasing.

It should be noted that the case studies were only conducted in a limited number of hospitals. Specifically, the pilot hospital in Indonesia is a specialty hospital (National Brain Center) and it may have different selection priorities than other hospitals in the country.

Notwithstanding the limitations, we consider the publication of the preliminary learnings as important to support others in planning their implementation projects as well as strengthening the ongoing projects themselves.

## Conclusions

By collecting and analysing the experiences of stakeholders across two different implementation case studies relating to drug purchasing on hospital level in Thailand and Indonesia, a set of measures was identified which could help to overcome important barriers for the implementation. The measures target challenges in terms of utility, methodology, data requirements, capacity building and training as well as the broader societal impact. In addition, it is recommended to carefully plan the implementation and organizational change to ensure commitment and fit to local context and culture.

A well-designed and collaborative change program for the implementation of MCDA-based purchasing will enable healthcare stakeholders to maximally benefit in terms of quality and effectiveness of care and access for patients.

## Supplementary Information

**Additional file 1**. Interview questionnaire.

**Additional file 2**. Summarized Responses for Thailand.

**Additional file 3**. Summarized Responses for Indonesia.

**Additional file 4**. Completed COREQ checklist (COnsolidated criteria for REporting Qualitative research).

## Data Availability

All summary data are available in the manuscript or the additional files. Additional anonymized individual responses may be accessed through request to the corresponding author.
