# Supplementary material for: Case studies for implementing MCDA for tender and purchasing decisions in hospitals in Indonesia and Thailand
Source: J Pharm Policy Pract. 2021 Jun 14;14:52. doi: 10.1186/s40545-021-00333-8 (PMC8200782; doi:10.1186/s40545-021-00333-8)
Supplement: Supplementary file 1 — Additional file 1. Interview questionnaire. [file 40545_2021_333_MOESM1_ESM.docx]

Questionnaire: Experience with MCDA Pilots

Discussion Guide

# Discussion with

| Name: |  |
| --- | --- |
| Organisation: |  |
| Function: |  |

| ***Choose all that are relevant*** | |
| --- | --- |
| **Professional education:**  Medical doctor  Pharmacist  Business / Finance  Nursing  Sciences / Research  Health-economist  Patient advocate  Other: _____________ | **Working at**  Hospital: _________  Tender agency  Research  Ministry of Health  Insurance  Industry  Academics    Other: _____________ |

***Introduction to interview:*** You have been nominated for this interview because you have been an important contributor to an Multi criteria decision analysis (MCDA) or performance-based decision initiative in your country. With this research, we aim to find out what the experiences of professionals like you were in the process and what we can learn from these experiences for the work in your country and for similar work in future.

We will record this interview for our own documentation and to enable us to confirm your answers in case of uncertainty. The recordings will not be published. We may cite specific wordings but only with your consent.

# Background information:

1. Can you define what MCDA is and what it is or could be used for in your country or organization?
2. Have you actively participated in work related to an MCDA pilot project in your country?
3. What was your role / contribution in this project(s)?
4. Do you have other practical experiences with MCDA? If yes, please describe.
5. What is the current or expected level of use of MCDA in healthcare policy or purchasing decisions in your country or organization?

**National level**:

**Hospital level**:

1. In your opinion, why should MCDA be used in your or any healthcare related organization?

# Pilot Implementation:

1. What difference do you expect the pilot to make in your organization?

| **Difference** | **Why** |
| --- | --- |
|  |  |
|  |  |
|  |  |
|  |  |

1. What went well up to now?
2. What were the difficulties up to now?
3. Have all stakeholders been involved who should have been involved?

# Broader MCDA Implementation:

1. How important are the barriers below for successful implementation of MCDA? Please rate between 1 (not important) and 5 (very important). Please explain the most important ones.

| **Barrier** | **Rating** | **Why** | |
| --- | --- | --- | --- |
| Change of process |  |  |  |
| Need for communication |  |  |  |
| Different / conflicting expectations |  |  |  |
| More work (perceived) |  |  |  |
| High transparency perceived as threat |  |  |  |
| Many stakeholders |  |  |  |
| Lack of training |  |  |  |
| Lack of experience |  |  |  |
| Conflicting interests |  |  |  |
| Perceived higher cost |  |  |  |
| Lack of political decision maker buy-in |  |  |  |
| ? |  |  |  |

1. In your opinion, which factors will be accelerating / facilitating the broad implementation of MCDA?
2. In your opinion, what will delay or inhibit the broad implementation of MCDA?
3. Who / which are the stakeholders who should participate in the process?
4. In your opinion, can patient organizations or advocates be (supportive, useful) part of MCDA initiatives and if so, how?
   - Examples?
5. Do you have any additional remarks?
