# Supplementary material for: Case studies for implementing MCDA for tender and purchasing decisions in hospitals in Indonesia and Thailand
Source: J Pharm Policy Pract. 2021 Jun 14;14:52. doi: 10.1186/s40545-021-00333-8 (PMC8200782; doi:10.1186/s40545-021-00333-8)
Supplement: Supplementary file 2 — Additional file 2. Summarized Responses for Thailand. [file 40545_2021_333_MOESM2_ESM.docx]

Summary of Interview Responses for the Hospital Pilot Study in Thailand

| Country Pilot | Thailand / Hospitals | | | | |
| --- | --- | --- | --- | --- | --- |
|  | ***Function*** | | | ***Role in Pilot*** | |
| Perspective | 1. Academic advisor; Ministry of Health 2. Academic advisor 3. Veterans General Hospital, Inventory management, Drug selection and procurement 4. Industry | | | 1. Leader: support and facilitate all the projects to be done correctly and with evidence-based evaluation. 2. Co-Lead of pilot implementation in hospitals; Principal investigator (PI) 3. Hospital owner of the process in purchasing department 4. Sponsor and facilitator | |
| MCDA Definition | Multi-Criteria Decision analysis (MCDA) is a methodology for evaluating multiple diverse criteria in decision making based on evidence.  In Thailand, MCDA is applied to 4 main activities which are   1. Hospital drug procurement under Public procurement law 2. Pharmaceutical pricing or National tender and bidding 3. National formulary essential medicine listing (NLEM) and 4. Management of national health security fund (NHSO)   The approach is currently known as price performance evaluation. The difference between price performance and MCDA pilot is:   - % weight of criteria and relative weight of categories Quality / Non-Quality / Price: e.g. Price-performance 40 / 20 / 40 and MCDA 40 / 30 / 30 - the MCDA pilot has more scoring categories in quality + non-quality (ie. The rating becomes more granular with e.g. 5 levels instead of 3 levels) - different non-quality criteria (quality criteria are the same) | | | | |
| MCDA Experience | 1. Participated on all 4 levels listed above 2. Was consultant for tender bidding for government hospitals 3. Led pilot project with PAT 4. In gov’mnt procurement through tender and bidding, price performance is used to identify which company at what price level   HTA evaluation of new drugs  Experience since 2017; I was actively involved in developing the price-performance evaluation system. After government procurement had announced the new procurement act in August 2017 our team for hospital procurement struggled because we did not have the appropriate knowledge. We invited expertise from other hospital to advise and help to develop a price performance instrument which was then accepted by the hospital administration. We collected experience over three years now, gained better knowledge and confidence in using it.  As pharmaceutical company, we are actively bidding for multi-source products. It is in our interest to educate the hospitals for doing this methodologically better and transparently. | | | | |
| Expected National MCDA Use | The MCDA is currently used for the final evaluation and decision in the benefit package.  An increased of MCDA in pool purchasing on the national level is expected. Currently, price is still the predominant decision criterion. | | | | |
| Expected Hospital MCDA Use | almost, all hospital used MCDA (price performance) in procurement of products that have quality concerns such as CVS, CNS products | | | | |
| Rationale for using MCDA? | Where the quality assurance cannot be guaranteed on a national level, MCDA is needed to improve the quality at reasonable cost. By using MCDA, we can balance affordability with factors such as pharmaceutical quality, drug safety, level of interchangeability, and real-life outcomes to get the optimum choice for what we are willed to pay.  By using the criteria, we achieve transparency and we can check it according to the agreed criteria. In Thailand, we are concerned about transparency in the decisions.  For hospitals, which used the price performance evaluation before, the difference is mostly, that there is more consistency how the evaluation is done. | | | | |
| Expected Differences due to MCDA |  | | | | |
| Better patient health (3) | If patients receive better-quality medicines, it will be better for their health through improved safety, decreased ADRs, and improved effectiveness. | | | | |
| Transparency (2) | Clear documentation of decision based on the criteria | | | | |
| Better accuracy / consistency in scoring (2) | More rational, more consistent, more acceptable because of the process of introducing the tools including training and group agreement. | | | | |
| Better acceptance of purchasing decision by HCP and patients (2) | If there is a better acceptance by stakeholders such as prescribing physicians and patients, a better use of the medicine can be expected (adherence). | | | | |
| Quality improvement (1) |  | | | | |
| More fairness in tender (1) |  | | | | |
| Change in hospital purchasing practices (1) | When the hospitals have been trained and participated in the pilot, it is much easier for them to use price-performance evaluation | | | | |
| Better performance of product (1) | They (the purchasers) not only look at lowest price but also other performance criteria | | | | |
| Awareness | Everybody can better understand what is important in the selection of the drugs in the hospital | | | | |
| Better guidance of companies what type of products are expected (1) | If criteria are applied consistently across hospitals, suppliers will learn to give the required information | | | | |
| Better access for high-quality products (1) | Improved market conditions for those companies, which have high quality products | | | | |
| What went well? |  | | | | |
| Good communication / collaboration between project leaders and hospitals (2) | Collaboration and knowledge sharing between hospital and academic project leaders. If there was a barrier, they also went into the hospitals and supported them. | | | | |
| MCDA adoption (1) | Some of hospitals decided to change to using the new MCDA | | | | |
| Participant motivation (1) | all hospitals tried to learn about the project and MCDA method. Even if they only did it theoretically, they tried to do and understand it well. | | | | |
| Preparedness – it fits current process (1) | In most hospitals, they already used similar criteria – e.g. quality related. That makes it easier to explain the concept and methods to them. | | | | |
| What were the difficulties? |  | | | | |
| Criteria difficult to assess (all 4) | The difficulty with the MCDA pilot is that the measures can be interpretated differently by different pharmacists depending on which expertise they have. For example, with BE.  Non-Quality criteria: hard to find the evidence to scoring; depends on different hospital and setting | | | | |
| Standardizing the scoring (1) | (Practitioner) | | | | |
| Resistance to change (2) | In some hospitals, they never try to adapt and change to MCDA | | | | |
| Need for improvement of criteria (Practitioner) | MCDA needs to be adapted before to be used in real. For example, the assessment of macro-economic benefit is still quite subjective. Those criteria need to be better defined for them to use them consistently. | | | | |
| Practice integration (1) | Co-Lead | | | | |
| Need for training (1) | (practitioner) | | | | |
| Stakeholders |  | | | | Included |
| Hospital pharmacists |  | | | | Yes |
| Head of hosp. pharmacy |  | | | | Yes |
| Head of specialty department for PCT |  | | | | Yes |
| Director of hospital |  | | | | Yes |
| Academic pharmacists |  | | | | Yes |
| Procurement pharmacists |  | | | | yes |
| Prescribers / Clinicians | Physician who prescribes the products | | | | not |
| Pharmaceutical industry | (because they need to send the data for the evaluation)  In some hospitals, they involved pharmaceuticals companies after the project was initiated because they wanted companies to submit the data. | | | | Not or too late |
| Finance | (In our case, finance was contacted after the criteria were decided because they really don’t have any influence on this except for processing the decisions) | | | | not |
| FDA / Pricing agency | FDA registration committee and committee for reference price should join in MCDA workshop. There is some difference between the standard of registering process and drug purchasing in the hospital. Moreover, Reference price set as the budget of drug purchasing changed frequently and affects annual budget frame. | | | | not |
| **Barriers** | Mean | SDev |  | | |
| Change of process | 4.25 | 1.30 | At the beginning, change is a high barrier. But once they learned it and did it, it gets easier. For those who knew price performance evaluation, it was easier. However, taking only price is much easier. Everybody needs to be on board (e.g. hosp. director) | | |
| Need for communication | 4.00 | 0.71 | All stakeholder at every step should know and understand the same way (pharmacists, doctors, sales reps) | | |
| Different / conflicting expectations | 3.50 | 0.87 | It takes time to discuss and agree. Pharmacy, doctor, and drug company often have very different expectations. | | |
| More work (perceived) | 4.50 | 0.87 | Reading the submitted documents is a high work burden*  *a workload survey after the new procurement methods were introduced elicited complaint that many documents had to be read for the evaluation. | | |
| High transparency perceived as threat | 3.00 | 0.71 | Higher transparency is an advantage in Thailand, not a barrier | | |
| Many stakeholders | 3.75 | 1.30 | Internal and external organization | | |
| Lack of training | 5.00 | 0.00 | Many people don’t know the technical terms and scoring is difficult for them | | |
| Lack of experience | 4.00 | 1.00 |  | | |
| Conflicting interests | 3.75 | 1.30 |  | | |
| Perceived higher cost | 3.13 | 0.54 | Opportunity cost (time), wages (hours of work), documentation | | |
| Lack of political decision maker buy-in | 3.88 | 0.89 | If the hospital administration agrees or requests it, it is easy to progress | | |
|  |  |  | | | |
| Accelerators |  | | | | |
| Education on evaluation (3) | Training on how to evaluate and scoring from the documents:  It is difficult for the pharmacists to evaluate the documents submitted by the companies. They do not have the right education. Training is important and some supporting techniques, e.g. checklist  Some hospitals already use their own criteria, similar to the new concept. So, they are used to this. However, the scoring is difficult for them (new) | | | | |
| Support / advice (1) | A national institution or department who can support this if there are difficulties | | | | |
| National policy (3) | National Policy. If the MCDA is required by law, it will be more effective. The hospitals adhere to the law and regulations | | | | |
| Clarity of criteria (1) | Clarity in the criteria. Detailed instruction for each category | | | | |
| Simple Tools (1) | User friendly tools that make the use easier | | | | |
|  |  | | | | |
| Inhibitors |  | | | | |
| Lack of personnel (1) | Lack of manpower and engaging the stakeholders | | | | |
| Lack of education (1) | Lack of Educations of all stakeholders | | | | |
| Adverse hospital priorities (1) | hospital policy and hospital committee | | | | |
| Resistance to change / Stickiness of old practice (2) | With older pharmacists who have been longer in their job, it was sometimes difficult to change the practice or to learn the new method.  - Most pharmacists do not feel confident to use the new MCDA system in real practice because it is new / unknown / not yet validated. So, they continue to use the old criteria. | | | | |
| Lack of understand of methods (1) | Lack of understanding of the MCDA concept and method | | | | |
| Uncertainty / fuzziness of criteria (1) | The criteria scoring is still a bit too ‘fuzzy’ and needs to be better defined to improve the consistency among different raters | | | | |
| Distrust in manufacturer-submitted data (1) | Can the data be used for the evaluation, that have been submitted by the manufacturer? The manufacturers just submit a pile of paper and it would be a lot of extra-work to read through all of them. Any information collected form the manufacturer should be very concise. | | | | |
| Role of PAG's | - Advocate for the importance of product quality and high-quality products; increase awareness on the issue of poor product quality (towards the government) and that only price is not good. - NGO’s have influence in Thailand. They are part of the committee. They have the power to include new interventions in benefit package. (Note by APH: this is more relevant to high cost / innovative medication) - If PO were involved, they could better agree on the criteria. They could have interest in some of the criteria – like adverse events, packaging, leaflet | | | | |
| Additional remarks | - All hospitals commented that there should be a center in MoH that did the quality evaluation of the MCDA on national level. Only the price and non-quality criteria could then be evaluated by the hospital. - The weighting of the criteria should be dependent on the type of products. With more complex products non-price criteria should have a higher weight of than with products of low complexity. E.g. biosimilars | | | | |
