# Supplementary material for: Case studies for implementing MCDA for tender and purchasing decisions in hospitals in Indonesia and Thailand
Source: J Pharm Policy Pract. 2021 Jun 14;14:52. doi: 10.1186/s40545-021-00333-8 (PMC8200782; doi:10.1186/s40545-021-00333-8)
Supplement: Supplementary file 3 — Additional file 3. Summarized Responses for Indonesia. [file 40545_2021_333_MOESM3_ESM.docx]

Summary of Interview Responses for the Hospital Pilot Study in Indonesia

| **Country Pilot** | **Indonesia / Hospitals** | | | | |
| --- | --- | --- | --- | --- | --- |
|  | ***Function*** | | | ***Role in Pilot*** | |
| Perspective | - Academic pharmacologist - Hospital Director - Hospital Pharmacology & Therapy Dept. - Industry | | | - Project Lead - As Leader of pilot hospital - As hospital expert in pilot - Industry | |
| MCDA Definition | - MCDA is an objective method to select the best Off-Patent-Pharmaceutical Alternative for a national or a hospital formulary based on several criteria and indicators. - The choice of the criteria depends on the availability of the data for those criteria | | | | |
| MCDA Experience | Varying; most experience with industry expert and project Leader | | | | |
| Expected National MCDA Use | Particularly useful in objective drug determination, but not easy to implement on national level. Since 2014, by decree, there is an establish process for open tender for off-patent pharmaceuticals. This is for about 1000 items and concerns government and non-government hospitals. Here, the decision is mostly based on price. This may change but will take time. | | | | |
| Expected Hospital MCDA Use | Everything beyond or out of the government catalogue is procured by each hospital themselves or by hospital groups (“Manual purchasing”). MCDA may be a useful tool, but will have to be adjusted. The system has been elaborated for a private hospital (RS PON) and will be piloted for tendering in practice in the end of 2020 or beginning of 2021 | | | | |
| Rationale for using MCDA? | In the hospitals, MCDA will help to ensure that only the best drugs are listed. Physicians want all medicines and MCDA will support the pharmacy director to reduce the list through a rational selection process to the most effective and affordable products. Thereby, more criteria beyond efficacy and safety are considered, which are related to the needs of the hospital (price, quality, supply reliability, etc.). The second important goal is to minimize drug shortages. | | | | |
| Expected Differences due to MCDA |  | | | | |
| Transparency in tender or formulary selection (2) | Now only price. Other considerations are not transparent to industry. | | | | |
| Fairness in OPP market (1) | High quality manufacturers cannot compete on price only | | | | |
| Quality of products (4) | Maintain a good quality standard for the hospital formulary beyond the evaluation of BPPOM; | | | | |
| Better decisions on the formulary (1) | Pharmacist can screen the submissions of the clinicians and then choose the best medicine (quality and affordable) | | | | |
| Affordability (2) | Not always the cheapest but the best at an appropriate and relatively acceptable price. | | | | |
| More objective / rational decision (2) | Decisions more based on data / facts (equivalence, formulation etc.) | | | | |
| Better (monitoring of) availability (1) | Monitoring of availability and considering for future decisions | | | | |
| More (objective) consideration of value-added services (1) | More uniform assessment of the value of value-added services | | | | |
|  |  | | | | |
| What went well? |  | | | | |
| Creating interest in hospital (3) | participating pharmacists were convinced and thought that the criteria are immensely helpful for making a better selection. | | | | |
| Creating awareness (2) | Increased awareness on the issues and the potential solution MCDA both on policy level and hospital level. Already after the first workshop, the pharmacists started to be more critical concerning the criteria and asked for more documentation to be submitted. | | | | |
| Stakeholder involvement (1) | A stakeholder mapping helped to identify the relevant hospital participants (including hospital director, who is important for top-down realization) | | | | |
| Using local data (1) | As first step, hospital data were analysed to show to the hospital how MCDA could improve their decision making | | | | |
| What were the difficulties? |  | | | | |
| Getting the data for evaluation (2) | Pharmacovigilance is complex; it is not fully reported in Indonesia and difficult to differentiate by product; patients may not report side effects / adverse events to the hospital.  Data on macro-economic benefit is limited.  The distributors or manufacturers don’t always have the required information. We must educate the manufacturers to be able to supply the information. | | | | |
| Perception of being more complicated and more work (2) | Initially, the hospital pharmacists felt, this makes their job more complicated (more steps in the decision process). | | | | |
| Pandemic interrupted the initiative (2) | Because of pandemic, the communication between academics and hospital has become limited. Within hospital, there were other priorities which interrupted the piloting. | | | | |
| Drug shortages can have other reasons (1) | Reliability of drug supply: It is important to see why this happened. Sometimes, it is not the fault of the manufacturer. If the hospital suddenly needs much more of one drug and manufacturer cannot supply, the manufacturer (eg. in pandemic) did not do anything wrong. | | | | |
| Stakeholders |  | | | | Included |
| Academic leaders | Academics need to lead the initiative as neutral people to leverage the concept | | | | Yes |
| Hospital director | It is important to have the hospital leadership on board to get her/his support; if they are not aware, they don’t see the benefits but only additional work and cost. | | | | Yes |
| Hospital administration | They need to be aware to support the process | | | | Yes |
| Clinicians / Prescribers | The doctors need to know about the reasons for deciding on the drug formulary; they should know the reasons for the choices. They will be more appreciative and supportive if they understand this. | | | | yes |
| Pharmacists (supply manager) |  | | | | Yes |
| Therapeutic committee |  | | | | Yes |
| Hospital association | If the hospital shares the formulary, then a hospital association should be involved | | | | no |
| local FDA | The hospital stakeholders are not aware of some of the issues, eg. with pharmacovigilance. But with local FDA participation, they will get additional information from a trustworthy source. | | | | no |
| Pharmaceutical companies | the manufacture must be informed. Only if they know, they can give the necessary data to the hospital; directly or through distributor. | | | | no |
| Financial & legal department of hospital | The necessary financial information relating to product price was retrieved through the participating pharmacists.  But Legal & Finance may be involved in making the final decision with the hospital director; if they heard the discussions directly, they would realize better how this helps to enhance the hospital at affordable cost. | | | | no |
| Patient organization | A patient organization could play a role through specific focus group discussion or in-depth interviews to better understand the qualitative input from them. It would help to clarify what is important from their side. | | | | no |
|  |  | | | |  |
| **Barriers** | Mean | SDev |  | | |
| Change of process | 3.25 | 0.83 | The process for MCDA appears long (intensive) – it could work better if the process was simplified. How difficult the change is, depends on the individual hospital. Old habit will be hard to stop. | | |
| Need for communication | 5.00 | 0.00 | MCDA requires information on a range of factors (criteria) which must be retrieved from various other internal or external sources.  All stakeholders should be onboard and aligned, and avoid misunderstanding with hospital management. | | |
| Different / conflicting expectations | 4.13 | 0.74 | Not all the people have the same perception of what is best. Conflict might come up if individual preferences can no longer be realized. | | |
| More work (perceived) | 4.00 | 0.71 | MCDA needs a lot of documents, work, and consensus building before reaching a conclusion | | |
| High transparency perceived as threat | 3.75 | 1.30 | Transparency is an advantage for the hospital, because they can much better document that the decision was structured and not based on individual preferences or corruption. However, specific individuals may perceive this as a threat. | | |
| Many stakeholders | 2.25 | 0.83 | This is a big problem across hospitals, because in each district the different type of hospitals with different selection approaches causes a big variation in access to medicines. Within the hospital, it should not be such a high barrier. | | |
| Lack of training | 2.75 | 0.83 | There is a high variability in the capabilities. However, it is only a limited number of people (formulary) who need to be trained intensively. | | |
| Lack of experience | 3.50 | 0.50 | Only few pharmacists know about this or have experience in it, currently. In the longer term, they will have the experience. | | |
| Conflicting interests | 3.00 | 1.41 | It is one key objective to overcome the subjectivity. | | |
| Perceived higher cost | 2.25 | 1.09 | Cost is a problem because if the budget is increasing too much. If hospital chooses the more expensive brand, they have a problem. But with increasing transparency, competition, and regulation, manufacturer adjust their prices in reference to the cheapest. | | |
| Lack of political decision maker buy-in | 3.00 | 1.22 | This is more a problem on the national level than on the hospital level. For the hospital, the buy-in from the leadership is essential. | | |
|  |  |  | | | |
| Accelerators |  | | | | |
| Leadership support (2) | If the hospital director understands it and shares the vision for good outcomes, it is easier to implement the MCDA approach. | | | | |
| Training (3) | Training & awareness, knowledge of the tool for decision making. It should be part of continued training courses (CME) for professionals making these decisions in hospitals. This way, more people would know about the tools and the need for doing this. | | | | |
| Practical experience (1) | opportunities for applying it will help people to better understand how to do it. | | | | |
| Medical association and  Hospital association support (1) | Clinical specialist associations and hospital associations could be helpful / impactful for increasing the awareness and the acceptance of MCDA. | | | | |
| Using local hospital data (1) | If it can be shown in the local data, what the issue is and what difference could be made, it is much more convincing than some third-party examples | | | | |
| Finding solution for conflicts in interest (1) | If there are positive experience, how conflicts can be resolved, this will set a positive example | | | | |
| Access to data (1) | Access to data and data collection   - Prices of drugs - Enhanced quality documentation of the drugs (equivalence, reliability of drug supply, pharmacovigilance) - Data on economic outcomes | | | | |
| Awareness, common understanding | Create aligned awareness about issues and solution among all hospital stakeholders | | | | |
| Inhibitors |  | | | | |
| Lack of training and knowledge (3) | Drug procurement is not simple. It includes a lot of discussion with BPPOM/MoH and open tender with LKPP (national formulary) and good understanding of the criteria and scoring. | | | | |
| Doesn’t fit to current process (2) | In some cases, there may be a lack of understanding how to apply it in the hospital team or too much change of process is difficult | | | | |
| Industry rejection (1) | If there is too much push-back from the industry or too many manufacturers are excluded from the bidding, this will negatively impact the adoption | | | | |
| Complexity of MCDA process (1) | The process itself is quite laborious: it needs a lot of work and discussion; including pharmacists and clinicians. They are very busy and it is hard to get them to the table. | | | | |
| Lack of alignment (1) | Everyone must be in alignment when applying MCDA. It is because the process will change mindset, paradigm, and will create conflict interest | | | | |
| Role of PAG's | - Opportunity: Patients could share their experiences in the beginning of the process: focus group discussions, in depth interviews, questionnaire, qualitative research to conclude patient needs; this would help to convince some of other stakeholders. However, they would have to understand the principles and clinical background (education required) - Concern: patient involvement may impact the objectivity negatively | | | | |
