# Supplementary material for: Case studies for implementing MCDA for tender and purchasing decisions in hospitals in Indonesia and Thailand
Source: J Pharm Policy Pract. 2021 Jun 14;14:52. doi: 10.1186/s40545-021-00333-8 (PMC8200782; doi:10.1186/s40545-021-00333-8)
Supplement: Supplementary file 4 — Additional file 4. Completed COREQ checklist (COnsolidated criteria for REporting Qualitative research). [file 40545_2021_333_MOESM4_ESM.docx]

Completed COREQ checklist

COnsolidated criteria for REporting Qualitative research (Tong, Sainsbury, and Craig 2007)

# Domain 1: Research team and reflexivity Personal Characteristics

1. Interviewer/facilitator

Which author/s conducted the interview or focus group?

All interviews were conducted by Anke-Peggy Holtorf.

This is described in the methods section **(manuscript, line 138)**

2. Credentials
What were the researcher’s credentials? E.g. PhD, MD

Dr. rer. nat, Dipl. Biol., MBA **(manuscript, line 138)**

3. Occupation
What was their occupation at the time of the study?

Managing Director and Analyst at Health Outcomes Strategies GmbH; Adjunct Faculty at the University of Utah, College of Pharmacy

(**indicated in the manuscript, title page**)

4. Gender
Was the researcher male or female?

AP Holtorf is female (all interviews)

**(manuscript, line 138)**

5. Experience and training
What experience or training did the researcher have? Relationship with participants

Natural Sciences, Health economics & health policy, Previous research involving interviews.

The interviewer had no relationship to the interviewees; the project leaders in both countries knew the interviewer through previous workshops, publications, or collaborations. There was no pre-existing relationship or collaboration with the other interviewees.

**Not included in manuscript as not relevant to study.**

6. Relationship established
Was a relationship established prior to study commencement?

The interviewees were first contacted by intermediates known to them (local experts from pharmaceutical industry – industry coauthors) for their interest in participating in the interviews. (**see manuscript, line 128-131, 136-137)**

If they agreed to participate, the interviewer scheduled a time-slot for the interviews via an E-Mail. The interview questions were sent out to all registered participants at least 3 days before the interview by the interviewer. At the beginning of the interview, the interviewer explained again the purpose of the interview and confirmed the willingness to participate (**see manuscript, line 138-141)**

7. Participant knowledge of the interviewer
What did the participants know about the researcher? e.g. personal goals, reasons for doing the research

The interviewer was introduced via E-Mail by name to the participants when the contact between the interviewer and the participants was established.

They received an explanation of the interview objectives and process when initially contacted and asked to participate. The interviewer introduced herself at the beginning of the interview.

**N/A in manuscript**

8. Interviewer characteristics
What characteristics were reported about the interviewer/facilitator? e.g. Bias, assumptions, reasons, and interests in the research topic

The interviewer introduced her academic background and interests in the subject at the beginning of the interview.

**Introduction in Supplementary File 1**

# Domain 2: study design. Theoretical framework

9. Methodological orientation and Theory
What methodological orientation was stated to underpin the study? e.g. grounded theory, discourse analysis, ethnography, phenomenology, content analysis

The interviewees were informed that the content of their responses would be evaluated for revealing potential commonalities and differences. They were also informed that it was planned to publish the findings in an international peer-reviewed journal and that their name and contribution through the interviews would be acknowledged in the publication. **(manuscript line 131-133).**

After the interviews, the responses were analyzed for their content. The number of mentions were counted for most questions. The average rating of the barriers was calculated using an excel spreadsheet **(manuscript line 144-149).**

## Participant selection

10. Sampling
How were participants selected? e.g. purposive, convenience, consecutive, snowball

The participants were selected for their leadership in developing and implementing the MCDA tools in their respective environments and their willingness to participate (purposive convenient sample). (**manuscript, methods section, line 136**)

11. Method of approach
How were participants approached? e.g. face-to-face, telephone, mail, email

The original contact was established via telephone or E-Mail.

The interviews were scheduled electronically.

The interview was conducted by web-conference (GoToMeeting).

**(manuscript, Line 138-139)**

12. Sample size
How many participants were in the study?

2 pilot participants and 6 implementors from 2 countries (4 per country)

(**manuscript, see 127-131**)

13. Non-participation
How many people refused to participate or dropped out? Reasons?

In addition to those implementors who were interviewed, 2 implementors from the national policy workshop in Indonesia were interviewed. However, the responses were not evaluated and not reported in the manuscript because the pilot phase has not yet been reached.

**N/A in manuscript as not relevant to the research and results**

## Setting

14. Setting of data collection
Where was the data collected? e.g. home, clinic, workplace

It was left to the interviewee when and where the interview happened. Some did it from their private office during their off-work time, some from the workplace during their worktime.

**N/A in manuscript as not relevant to the research and results**

15. Presence of non-participants
Was anyone else present besides the participants and researchers?

Participation of other people was not excluded but no additional people participated in the interviews or listened to them.

**N/A in manuscript as not relevant to the research and results**

16. Description of sample
What are the important characteristics of the sample? e.g. demographic data, date

We were looking for **active implementors** in each of the pilot implementation projects. All participants were known for their active engagement in their respective environment. They could be pharmacy specialists, hospital managers, or other healthcare policy stakeholders (**see manuscript, Line 136-137**).

## Data collection

17. Interview guide
Were questions, prompts, guides provided by the authors? Was it pilot tested?

The interview was first designed by AP Holtorf and critically reviewed by non-participating peers. It was then pilot tested in 2 interviews with pharmaceutical industry experts. (**Manuscript, Line 127-129**)

Only minor revision was deemed necessary after the piloting (e.g., wording; sequence of questions for better flow).

The pilot interviews served multiple purposes: 1.) to pilot the questionnaire, 2.) to train and standardize the interviewer for the interview flow, 3.) to lay the baseline knowledge for the country and pilot context for the interviewer.

(These last 2 sentences are not included in the manuscript but could be added if deemed useful additional information)

18. Repeat interviews
Were repeat interviews carried out? If yes, how many?

Each interview was individual and not repeated. However, the respondents received the interview notes and had the opportunity to add or change the contents as they deemed necessary. A few interviewees suggested minor corrections (language or clarification)

**(See manuscript lines 142-143)**

19. Audio/visual recording
Did the research use audio or visual recording to collect the data?

The screen of the interviewer was shared during the interview, and the interviewees could see the notes that were taken. The interviews were recorded (with agreement of the interviewees)

**(see manuscript line 141-142)**

20. Field notes
Were field notes made during and/or after the interview or focus group?

The notes were taken during the interview. Directly after the interview (within a maximum of 76 hours), the notes were ‘revised’ (correcting typing mistakes, formulating full sentences, deleting duplications, etc.) with the help of the recordings.

(**in manuscript, lines 141-143)**

21. Duration
What was the duration of the interviews or focus group?

All interviews lasted between 45 and 60 minutes (**see line 138**).

22. Data saturation
Was data saturation discussed?

N.A. as not relevant to the research and results

23. Transcripts returned
Were transcripts returned to participants for comment and/or correction?

Yes (see above) (**in manuscript, lines 141-143)**

# Domain 3: analysis and findings

## Data analysis

24. Number of data coders
How many data coders coded the data?

One (AP Holtorf evaluated and categorized all responses)

(**Manuscript, line 144-145**

25. Description of the coding tree
Did authors provide a description of the coding tree?

The approach to evaluation is explained in the **methods section
(manuscript, Lines 144-149)**

For the categories (coding), see results section (**Tables 1-3**)

26. Derivation of themes
Were themes identified in advance or derived from the data?

Derived from data **(manuscript, lines 144-149)**

27. Software
What software, if applicable, was used to manage the data?

Numerical evaluation was done with an excel spreadsheet.

(**Manuscript, line 147)**

28. Participant checking
Did participants provide feedback on the findings?

Interviewees provided feedback on the notes relating to their own contribution.

The overall findings were shared with the interviewees who lead the projects in their respective countries. They will be shared further immediately after publication.

**N/A in manuscript as not relevant to research and results**

## Reporting

29. Quotations presented
Were participant quotations presented to illustrate the themes/findings?
Was each quotation identified? e.g. participant number

No quotations are used.

30. Data and findings consistent
Was there consistency between the data presented and the findings?

Yes, the data presented in **Tables 1-3** and **Figures 1-2** represent the data collected during the interview. Categorization in Table 1-3 was derived from the responses.

**Table 4** interprets the findings in the context of published categories (Utility, Methodology, Data requirements, Capacity/training requirements, Broader societal impact) and **Figure** 3 puts the findings in context of Kotter’s change framework.

31. Clarity of major themes
Were major themes clearly presented in the findings?

Yes

32. Clarity of minor themes
Is there a description of diverse cases or discussion of minor themes?

Yes (see also **weighting analysis in Figure 2**). Generally, a large congruency was found among the responses of the interviewees and there was no important diversion. Differences depended on country context, position of the implementor, and experience of the implementor. Not all implementors had experience in all levels of implementation.

**Reference:** Tong, Allison, Peter Sainsbury, and Jonathan Craig. 2007. “Consolidated Criteria for Reporting Qualitative Research (COREQ): A 32-Item Checklist for Interviews and Focus Groups.” *International Journal for Quality in Health Care: Journal of the International Society for Quality in Health Care* 19 (6): 349–57. https://doi.org/10.1093/intqhc/mzm042.
